# ‘They’re Going to Die at Some Point, but We’re all Going to Die’ – A Qualitative Exploration of Bereavement in Later Life

**DOI:** 10.1177/00302228211053058

**Published:** 2021-11-25

**Authors:** Chao Fang, Sam Carr

**Affiliations:** Department of Education and Centre for Death and Society, 1555 University of Bath, Bath, UK

**Keywords:** bereavement, ageing, relational loss, existential distress, grief literacy

## Abstract

This article reports on a qualitative study to investigate what bereavement means to older people. Drawing upon 80 in-depth interviews collected from eight British and Australian retirement communities, our study revealed that facing bereavement while ageing includes experiences of losing both others and the wholeness of the self. Core themes identified how the experience of losing others can be compounded by ageing-related challenges, undermining older people’s defence from bereavement and frustrating their fundamental meaning and being. The older people’s dynamic responses were also captured, highlighting the importance of supporting their agency to deal with the deeper pain of loss. By extending the concept of bereavement in later life, we also called for a more grief literate culture to mitigate the multifaceted and often deeper distresses of bereavement that older people may face alongside ageing.

## Introduction

Bereavement is a challenging life event that may occur at any point across the lifespan. The confrontation of loss and bereavement is particularly prevalent in later life when people become more likely to experience a myriad of loss, such as that of a partner, siblings, friends and/or other family members (d’Epinay et al., 2003; Lekalakala-Mokgele, 2018). Such bereavement experiences can be deeply painful in a multitude of ways, as a result of losing relationships that have long shaped their everyday life and identities (Fang, 2020; Richardson, 2014; Larsson et al., 2017).

The disruptive and distressing nature of bereavement in old age may be further compounded by other ageing-related issues. That is, deteriorating physio-cognitive capacities and reduced social networks can undermine older people’s adjustments to an everyday life that is drastically impacted by loss (Lekalakala-Mokgele, 2018). The experiences of outliving their loved ones and other contemporaries may also contribute to older people’s awareness of separation from the external world and their own finitude, further undermining their motivation to rebuild their life after loss (Carr & Fang, 2021; Larsson et al., 2017). As such, experiencing bereavement alongside ageing may inextricably connect to a process of losing a taken-for-granted world, which has long helped older people retain resilience and (re)define the meaning of their identity and existence (Naef et al., 2013). In facing the accumulating loss of resources and coping mechanisms as part of ageing, bereaved people may be further thrust into existential fears of being unable to reaffirm themselves and find a new way of existing in an unfamiliar world (Ettema, et al., 2010; Larsson, et al., 2017).

The bereavement of older people has received broad attention thanks to the growing emphases on population ageing and wellbeing. Research into this topic has examined the experiences of losing a loved one, often a spouse or other close figures, from physical, psychological, social, practical, spatial and material perspectives (e.g. d’Epinay et al., 2003; Maddrell, 2016; Newson et al., 2011; Richardson, 2014; Utz, 2006). Despite the inter-disciplinary and in-depth insights into older people’s diverse bereavement experiences, most research focuses on bereavement in later life as a narrowly defined experience of losing others (e.g. attachment, social resources and physical intimacy). However, bereavement experiences may be compounded by ageing in ways that compromise older people’s identity and existence. To better understand bereavement in later life, it may be important to explore the deeper meaning of loss and how losing others in old age may propel older people closer to a loss of self.

To extend the conceptual boundaries of bereavement in later life, we report on a qualitative study, based on 80 in-depth interviews with long-lived people of a broad age range, residing in retirement communities in the United Kingdom and Australia. Drawing on this large set of qualitative data from older people with varied experiences of loss and bereavement, we sought to explore (1) what bereavement means to older people, (2) why and how bereavement may run deeper to threaten the integrity of older people’s ‘self’ and (3) how they respond to these existential challenges of bereavement as they age. To build upon this extended understanding, we sought to inform future practice to holistically support older people’s deeply painful experiences of bereavement alongside ageing.

## Understanding Bereavement in the Context of Ageing

Bereavement in later life has often been researched as a disruptive life event facing older people, which can thoroughly challenge many aspects of their lives and may prompt substantial readjustments (Carr & Mooney, 2021). An integrative review on spousal loss has demonstrated the variation and complexity of bereavement in old age:

‘[A] new identity as widow/er and striving for independence in the face of disrupted everyday activities and routines, loneliness, health concerns and changed relationships within the family and social network are essential features of older persons’ bereavement experience’. (Naef et al., 2013: 1108)

While studies have primarily focused on spousal loss, literature regarding the loss of children, siblings and friends among older people also illustrates bereavement as a multifacted disruption to everyday life (e.g. d’Epinay et al., 2003; Fang, 2020; Moss & Moss, 2003). This diverse focus on bereavement has further highlighted that older people may face different types of loss and even multiple concurrent loss of others alongside ageing (Lekalakala-Mokgele, 2018).

To capture the complex nature of bereavement in later life, studies have predominantly approached older bereaved people’s experiences from two main vantage points: outcomes and responses. By highlighting negative and/or pathological outcomes of grief, a large body of studies measured the associations between loss of others and an array of outcome variables (e.g. depression, isolation, increased commobilities and mortality) (Stroebe et al., 2007; Utz et al., 2012). In addition to highlighting the potentially detrimental impacts of loss, researchers have also explored bereavement as an embodied everyday experience of meaning reconstruction in response to the shattered life world and self-narratives (Neimeyer & Holland, 2015). Whilst ageing-related challenges to bereavement experiences (e.g. decline in health and social connection) were noted, these studies primarily focused on older bereaved people’s dynamic and diverse negotiations for meaning to make sense of their loss (Fang, 2020; Holm et al., 2019).

As discussed above, current studies on older people have largely mirrorored the broader picture of bereavement research, suggesting the disruptive nature of bereavement and the significance of resilience in facing the loss of others (Stroebe & Schut, 2010; Valentine, 2008). These studies, however, often focused on specific aspects of ageing (e.g. health, loneliness, dependence and old age security) alongside other non–ageing-related challenges (e.g. the nature of relationship with the deceased, family structure, gender and cultural norms). As such, little emphasis was placed on the pervasive impacts of ageing on older people’s bereavement experiences; how ageing may be ingrained into day-to-day life disrupting their resilience to reconstruct meaning, and how these experiences may further give rise to a deeper sense of losing the wholeness of the self. To better understand bereavement in the context of ageing, further insights from ageing-related studies need to be incorperated into bereavement studies of older people.

Compared to bereavement research, the social science of ageing studies has hinted at the enduring and deep impacts that ageing may have on older people’s negotiations for meaning in varied circumstances. It is worth noting that the healthy ageing paradigm has prevailed in current research, highlighting the importance of retaining older people’s autonomy and resilience in all aspects of their lives, namely, physical, psychological, social and spiritual (e.g. Hillcoat-Nallétamby, 2014; Sadler & Biggs, 2006). Research has also suggested that ageing may lead to a transition when facing growing challenges of dependence, frailty and isolation due to bodily deterioration, immobility, bereavement and terminal illness (Nicholson et al., 2012). These changes and losses alongside ageing may undermine older people’s capacity to retain meaning and strength (Ettema et al., 2010).

In facing the accumulation of losses and challenges alongside (or as a result of) ageing, older people may be intimately connected to a gradual process of losing *defence* to cope and adapt to disruptive circumstances. Such diminishing defence has been observed in the contexts of advanced old age and at the end of life ‘[w]hen finitude or impairment terminates the possibility of cherished self-promises to… put things right’ (Johnson, 2013: 185). Given the potentially deteriorating nature of ageing, these experiences may not only be restricted to highly fragile phases of old age but also over the broader course of ageing. By gradually losing a healthy body, meaningful social networks and the continuity of life, older people may suffer from decreased capability and motivation to draw on meaningful resources to defend against disruptive challenges.

This decline in defence may also entail a deeper dimension, where older people feel they have less control (agency) to define their lives and may further experience growing pains of being forgotten, unimportant and irretrievably lost in the past (Bolmsjö et al., 2019; Carr & Fang, 2021). This deeper pain may be further compounded by a lack of literacy for their loss and grief (e.g. emotional language, interpersonal support and social values), leaving older bereaved people with few meaningful resources and channels to obtain understanding and support, especially when facing other ageing-related challenges (Breen, et al., 2020; Sjöberg, et al., 2018).

The experience of ageing can undermine older people’s negotiations for meaning and identity in an unfamiliar world after loss and beravement (Neymier & Holland, 2015; Attig, 2010). Despite this, bereavement is yet to be closely examined in light of the findings captured by the above ageing studies. Given the risks of losing defence and confronting existential crises alongside ageing, it is also important and necessary to question, if and how bereavement may contribute to this deeply painful process of losing purpose and identity in the world, and how older people respond to the deeper impacts of their loss. By closely examining 80 older people’s accounts, our study sought to better understand the deeper meanings of bereavement in later life, and their unique needs and challenges.

## Methods

This article draws on in-depth interviews with 80 older people, which were conducted October 2019–February 2020, as part of a larger qualitative project investigating emotional loneliness in eight independent-living retirement villages in the UK and Australia. The study received ethical approval from the authors’ institution and also obtained permission from all of the participating retirement communities. The site managers acted as gatekeepers to facilitate contact with participants for eight trained researchers, four in the UK (including the authors) and four in Australia. Written informed consent was obtained from all participants.

Interviews with 80 socio-demongraphically diverse participants were conducted across England and in a metropolitan area of Southern Australia, including 55 women and 25 men (Table 1). The average age of the participants was 79 (*SD* = 7.6) with an age arrange between 55–93. This dataset provides a diverse sample regarding bereavement experiences: 45 participants had recently lost intimate loved ones (often a spouse/partner or a family member) while the remaining 35 individuals had not yet experienced significant loss and/or only lost comparatively distant others (e.g. friends, neighbours and former colleagues). This sample allows for greater insights into the scope of bereavement, which can refer to the experience of losing not only close others but also people in wider social circles. Despite this study’s situation within retirement communities, findings remain broadly applicable as we do not see this context as a unique environment that decisively shapes older people’s bereavement experiences. Rather, this unique context affords us an opportunity to explore how older people may conceive moving to community-living as a potential solution to defend themselves from bereavement in their ongoing lives.

**Table 1.** Socio-demongraphical Characteristics of Interview Samples.

Characteristics	Sample (*N* = 80)
Age (years)	
55–60	2
61–70	9
71–80	35
81–90	31
91+	3
Gender	
Female	55
Male	25
Marital status	
Divorced/separated	7
Married/partnered	26
Unmarried	4
Widowed/widowered	43
Health	
Has a chronic condition(s)	28
Healthy	52
Household	
With the spouse/partner	41
Living alone	37
With friends	2

The researchers conducted one-to-one interviews within the participants’ home. Adopting a semi-structured style with open-ended questions, the participants were invited to talk freely through their life histories, particularly, how their lived experiences shaped their emotional lives and how these were related to their retirement-living. Despite loneliness being a primary focus of the interview, experiences of loss and bereavement emerged naturally throughout the conversation. With support of the gatekeepers, a rapport with the participants was developed before the interview, enabling them to feel at ease to share their complicated experiences and often deeper feelings with the interviewers. The interviews lasted from 70 -200 minutes and averaged around 100 minutes. In total, approximately 8000 minutes of interviewes were audio-recorded and professionally transcribed.

All the interview transcripts were analysed thematically employing an inductive approach to interpret loss and other ageing-related challenges facing the older people and how they perceived and responded to these experiences. This analysis method enabled us to form our theoretical understanding of the deeper meanings of bereavement in later life by directly drawing upon vivid accounts and without being restricted by pre-existing frameworks (Mason, 2002). The two authors coded each interview transcript independently using a combination of QSR NVivo 12 and MS Word based reading and coding. We coded data into three broad categories - circumstance, experiences and responses of older bereaved people. Based on frequent meetings between the authors, these (sub)codes were further thematically grouped to illustrate how bereavement may run deeper to shapes older people’s lives.

## Findings

Four key themes were identified from the analysis, illustrating how bereavement in the context of ageing may be inextricably connected to: (1) losing relational being in everyday lives, (2) decline in defence, (3) deeper pain of losing the self and (4) striving to retain a sense of identity. Theme one underlined older people’s bereavement as a highly disruptive experience of losing others and relational being. Theme two captured how ageing-related challenges can undermine the capability to deal with relational loss. Theme three illustrated how bereavement can be intertwined with ageing to further give rise to existential loss of self. The final theme highlighted how they respond to the deeper pain of loss. To protect the participants’ confidentiality, pseudonyms were used throughout.

### Losing Relational Being in Ongoing Lives

Research has widely acknowledged beravement as loss of others, which can significantly disrupt the consistency and meaningfulness of older people’s everday lives (Neimeyer & Holland, 2015; Fang, 2020). Central to these challenges is the loss of a long-term relationship and/or concurrent loss of individuals that have shaped and affirmed their taken-for-granted lives and identities.

More than half of our interviewees had lost a spouse/partner after spending considerable time together. For many, such a loss could powerfully shatter their memories, habits and bonds intimately built with their loved one, challenging their sense of self in a relational sense (Bradley & Caffert, 2001). Paula felt she was nobody after losing her husband whom she had cared for, for more than 10 years:

Paula: “[W]hile he was alive and I was his full-time carer, companion, friend, we had a ball even though he was in a wheelchair, but when he was gone I didn’t know where I fitted anymore. I didn’t know who I was anymore because I wasn’t …”

The overwhelming sense of becoming a ‘nobody’ was also connected to losing the meaningful and enduring bonds that to her were irreplaceable. For these older people, their lives may no longer be complete without their spouse/partner. This pain was vividly captured by Simon after losing his wife:

Simon: “I knew her for 73 years she was part of my life. It’s like cutting off half of you. And we were, we were just one… [y]ou just forget for a moment, or when you wake up in the morning you think ah. Just for a second you’ve forgotten that she’s not there. I mean, eventually I suppose. But it will never be the same.”

The loss of a spouse/partner could further propel the older people towards a sense of loneliness in their ongoing lives. By losing intimate bonds (and possibly the associated social connections), they may experience a painful confrontation with themselves being cut off from their relational lives. Philip felt he was left alone after losing his wife:

Philip: “She died about four years ago, just over four years ago, yes. Then it was just me and the dogs and the fish. Yes… Take what comes. I shall be unhappy when these dogs start dying, very.”

Such loneliness may also have a physical component intimately connected to the embodied experiences of losing physical intimacy, that had previously affirmed the older people’s bonds with their loved one. For Philip, he missed a goodnight kiss and sex with his wife as part of his romantic life:

Philip: “I gave a brief resume of my wife [at the funeral] and I said, ‘I’ll mention this, kissing good night. I said the one thing I will miss is a goodnight kiss,’ and blow me… I suppose all my life ‘sex’ has been ‘lovemaking’. I mean, we are really getting personal now but when my wife died, I missed that so much.”

In facing spousal loss in later life, the older people may have lost both a loved one and/or the only intimate figure in their everyday lives. By having no-one-else to turn to both emotionally and physically, some older bereaved people (especially widows) found themselves feeling fragile and unstable, further giving rise to a sense of insecurity. After suffering a burglary and confrontation in her home, Jacqueline felt the fears of being a widow and living alone penetrated her everyday life:

Jacqueline: “Yes, it [the burglary] made me feel that way, that I had nobody else living in the house with me. I just had to rely on myself... Well, I felt very vulnerable after that. Any little creak or noise I heard.”

The impact of bereavement on the older people’s relational being was not only restricted to loss of closest others, as some felt that losing friends and other contemproraries could profoundly deprive them of relationships that they had long used to affirm their social identities. For Mary, she dreadfully missed her social life and the feeling of being a friend:

Mary: “I had a couple stayed for lunch about three weeks ago. That is the first time I’d put lunch on for somebody for a number of years, because they are all dead, they’ve gone. I’ve had loads of friends, dear, they’ve died.”

### Decline in Defence

Growing older does not necessarily result in losing the means to retain resilience in the face of bereavement. However, in facing bodily deterioration and the loss of other special resources (e.g. social connection, inner strength) alongside ageing, older people may confront increasing risks of losing agency and coping resources to defend from the void of others (Johnson, 2013; Sjöberg et al., 2018).

Studies have suggested that attachment and social connection can provide older people with an important buffer from bereavement in late life (Michael Bradley & Cafferty, 2001; Morgan et al., 2019). However, the older people in our study frequently revealed their concerns about their gradually diminishing social resources and channels to restore the meaning for the loss of others and relational being. Two contrasting pictures below highlight the risk of painfully losing social protection to deal with bereavement in the ongoing process of ageing. For Elizebeth, despite the shrinking of her network of widow friends, having regular gatherings with them significantly supported her grief:

Elizebeth: “There’s only five of us left now but we meet up for dinner, or lunch. That was good. Talk to them because they were all different but we would laugh, although you could cry, but we would turn that into laughing.”

Conversely, Lucy was saddened as she had lost all her friends on whom she relied for support in facing the ongoing struggles of losing her husband:

Lucy: “Yes. Friendships are difficult when you get older. The friends that I had in Newcastle, it was a case of, “Oh yes we’ll come and see you,” but they are all my age. We’ve all got the same problem. And to be honest, a lot of them have died… Really there is no one. There’s not many people left [to rely on], which is sad…”

In addition to the loss of social protection, the impact of ageing could undermine the older people’s motivation to actively cope with their bereavement. Whilst growing older, some participants conveyed frustration over their increased fragility, dependence and other concurrent losses, further highligting a sense of tiredness for life. Lucy, being in her late 80s, felt she had no energy left to cope with her loss. When asked if she was missing her late husband and friends, she said:

Lucy: “No… I find now I have to channel into survival.”

This tiredness could also weaken the older people’s resilience in facing the voids left by their loss. Moureen found that in reaching an old age, she had exhausted her mental capability to face her spousal loss, even remembering the duration of her marriage was extremely painful:

Interviewer: When did you get married?

Moureen: When I was 22.

Interviewer: Yes.

Moureen: And he died… Jonny, we call him Jonny, he died in 2016. So whatever that was.

Interviewer: A few years back.

Moureen: Don’t even say it, it’s just so many. Makes me sad, I’m very old. But it flies by.

This declining defence for bereavement in old age could be further compounded by a ‘stiff upper lip’, a mentality of lacking emotional language to express emotions and seek support. This inability to verbally express emotion was widely seen amongst the older people (especially men) in our sample, who lived through certain historical, cultural and familial circumstances where high levels of emotional control were required (e.g. wartime, postwar hardships and family conflicts). By disabling older people’s mental capability to acknowledge and cope with the pain of loss, such a ‘stoic’ approach could further undermine older people’s already deminishing defence for bereavement:

Simon: You had to keep a stiff upper lip as it was called and I suppose that’s stuck with me. I mean, people in my generations who came through the war, not that we were fighting, I was too young, a child, a boy, you had to get on with all sorts of things.

Interviewer: So you had to keep some emotions inside of you.

Simon: Yes. We’ve got troubles as well. We don’t want to hear all your troubles. Just keep it, get on with it. I mean, people do seem to collapse at the moment and you think what’s the matter, just sort it out.

Interviewer: Yes, exactly. So for you, you just try to face and deal with it on your own?

Simon Yes. I feel sorry for myself at times when you are sat and you think oh god, I do wish she [my wife] was here and I do miss her.

### Deeper Pain of Losing the Self

Facing bereavement in later life, as illustrated above, may closely connect to both the impact of losing others and also the increased difficulties in restoring meaning from the loss of lives and identities. These compounded challenges may accumulate and even intensify alongside ageing, gradually giving rise to a deeply painful process, in which the older people’s lives, memories and narratives may increasingly fade and eventually become irretrievably lost in the past (Ettema et al., 2010; Larsson et al., 2017).

Many older people in our study not only lost their loved one but also had to face a myriad of losses of people in their family and social lives as a result of ageing. By losing others who had participated in their lives, they could face bereavement as a deeper fear of being forgotten. Margaret described losing people who remembered her life as a painful threat to her being:

Margaret: “And the older you get, the more you like finding people who remember your parents and your home and your youth are gone, which is what I most fear about getting too old. …yes [emotional]…that all the people that remember any of that are gone”.

As Emma explained, the pain of loss could further plunge the older people into existential struggles of longing for the sense of self:

Emma: “Absolutely, and ‘who am I?’ This is why old people talk about their lives, their past, or whatever, because they want you to know who they really are inside of the old visage.”

Some even felt their lives no longer mattered as a result of losing people to share and reminisce about their lives with:

Harold: “My life? Nobody wants to know about my life!”

The impact of losing others in old age might not be restricted to the pain of being forgotten but could be intimately connected to an ontological realisation of the finitude of the self. The experiences of facing the death of a family member, a friend or even a distant peer may serve as a painful reminder of the older people’s own death. For Theresa, she was losing not only people she knew but also her own life:

Theresa: “Obviously, you know. They’re going to die at some point, but we’re all going to die, you just say; we’ll have to deal with that as we go along, yes. Yes, as we get older.”

For some older people, the death of comtemporaries could be particularly powerful in triggering the fear of approaching the end of life. After losing his friends, Robert painfully realised his own mortality as an inevitable part of growing older:

Robert: “I did sort of think, a couple of years back, ‘Oh, I’m now at the age that Jim died.’ But it’s one of the things that you come to learn, these things happen in life, I’m afraid… There was a young lady, younger than us, again died of cancer. That was one of our bridge group. We sort of knew it was going to happen, but it happened quicker than was expected, and again, that brings home your mortality.”

By gradually and often increasingly losing others and confronting their own finitude, the painful experiences of feeling forgotten and the fear of ceasing to exist could further prompt a growing sense of fading away. That is, the older people were likely to experience the impact of loss as a process of being fundamentally separated and alienated from others and the external world. Iris felt she was left alone in the world as her loved ones and other comtemporaries were no longer there to help her feel connected to the external world:

Iris: “The only other thing is of course that most of my friends are dead. I’m 90 on Christmas day and when I go through my life, my school friends, most of them are gone, my college friends, most of them are gone. I’m the only in-law left, I’m the only great grandparent left. So that aspect of the extended family becomes less and less… Yes, then it becomes lonely as regard your future and your past life and the people that I was familiar with, people that I worked with, of course, and the people that I had social contact with and relatives. My own family, I’ve only got one brother left and my husband was Dutch, and the Dutch family, one sister-in-law. So I’m finding that I’m standing alone as regards my former life.”

Conversely, concerns regarding fading away could also compound the older people’s deeper pain of bereavement. Susan described that she was tired of losing people and ‘ready to die’:

Susan: You know, underneath it all I wouldn’t mind leaving this world. Everyone has

died and I think I’m lonely

### Striving to Retain a Sense of Identity

Loss and bereavement in later life may more deeply challenge both their relational lives and the more existential dimensions of the self. However, the older people also demonstrated their longing for meaning to restore their defence and further to retain their sense of the self moving forward.

Many participants admitted that one of their primary motivations to move to retirement communities was to defend themselves from the deeply painful impacts compounded by bereavement and other ageing-related challenges. For some bereaved older people, moving to a new community-based environment was an attempt to mitigate the pain of losing others and further to reverse the process of their lives fading away. Paula experienced a dramatic transition from ‘letting go of life’ to opening ‘a new chapter’:

Paula: “So it took a while to, as I say, get the act together, which after four years I was thinking I’ve got to do something with my life and not just let it go and suddenly I woke up one morning and thought, right, this is it, let’s get the place [my former flat] cleared out, get the place painted and decorated and then we’ll see where we go… I have to see it [moving to the community] as a completely new chapter in my life.”

For some, community-living enabled them to restore their connections with others and the external world on a day-to-day basis. For Stephen, feeling connected and needed did not require something grand but started from small everyday activities, such as having meals:

Stephen: “The first six months after she died, I guess I was fairly lonely. The worst part was sitting down to a meal at night. Breakfast was okay, because we always scrapped for that. But to cook a meal and sit down on your own in the house, and I came here to be in a community. And I recovered, I think, because of the fact that I was in community with people here, I wasn’t on my own any longer.”

While seeking to restore their being within their community and wider society, the older people also actively negotiated deeper meanings to preserve the identity of their loved one(s) and themselves moving forward through inter-generational interactions. For Philip, sharing memories about his late wife with his granddaughter was a meaningful way to remember her and to foster continuing bonds:

Philip: I’ve got all these albums of photographs, I sit down and go through years of photographs and remember things between us and that helps, yes, in some ways. I mean, even Felicity, she did reply, I read the reply, she said she saw a heron and she associated that with Grandma, so I remind her about the Kingfishers and one thing and another. She answered that about it…”

For these older people, passing on memories about their loved one(s) to younger generations could also help them reaffirm their own lives and further to preserve their sense of being. As the only child, Pauline felt a profound need to remember her parents and her early lives:

Pauline: “I said to my elder daughter the other day, ‘I would give anything to have a chat about my mum and dad’.”

Further in facing the accumulation of the existential pain of losing others and themselves, some older people (especially those in advanced old age) managed to adjust their expectations about meaning in ongoing life to retain inner strength. When reflecting on his mother’s death, Adam, in his 80s, acknowledged the deeper meanings of loss and life:

Adam: So I think she had a good life. The modern way of funerals making it a tribute to someone’s life, if you know what I mean, rather than grief.

Interviewer: Yes, the acknowledgement of a life well lived is meaningful.

Adam: Yes, that’s much better. We all get to an age where you know you’re mortal and your time will come, and that’s part of this whole thing, isn’t it? You do this in order to live life to the fullest for as long as you can.

Despite the dynamic responses to restoring the deeper meanings from loss and bereavement in the context of ageing, these older people also confronted barriers in their communities and wider society. For example, Ian pointed out the lack of adequate mechanisms and knowledge in understanding and supporting the deeper pains of bereavement in the retirement communities:

Ian: “I don’t think the consequences of the move [into retirement villages] are appreciated enough by people moving nor certainly by the organisation, not least the string of bereavements that many people have to manage and the lack of anything formally organised to help with that process.”

Such lack of support could also be experienced in wider society as what Lucy called ‘rejection’, thus requiring a more grief-supportive culture to address older bereaved people’s needs for meaning and being:

Lucy: “There’s couples and as a lot of couples you do feel rejected… Some of them are very nice, but they don’t say, ‘Oh come and sit here. Come with us’. They’ll talk, but there’s a barrier.”

## Discussion

Our data provided insight into what bereavement means in later life, showing that it is not only an experience of dealing with the loss of others but may also be intimately connected to a gradual process of losing the wholeness of self. By interpreting vivid accounts of 80 long-lived individuals in the UK and Australia, it became evident that bereavement for many older people was not restricted to loss of closest others but may relate to the death of other distant people and contemporaries. We also found that these losses may compound the growing challenges of deterioration, frailty and isolation alongside ageing, further questioning the older people’s lives and meaning at a more existential level. As such, our study focused on older people’s experiences of loss in the context of ageing to explore the deeper meanings of bereavement.

Adopting a qualitative approach, our study reaffirmed the existing understanding of bereavement in old age, as a disruptive experience in which individuals can express resilience (e.g. Holm et al., 2019; Naef et al., 2013; Neimeyer & Holland, 2015; Newson et al., 2011). As demonstrated in our analysis, losing others and especially long-term intimate relationships could shatter the older people’s taken-for-granted lives and relational being in marital, social, emotional and physical (even sexual) aspects of their ageing lives (e.g. d’Epinay et al., 2003; Lekalakala-Mokgele, 2018; Richardson, 2014). Meanwhile, we also captured their resilient responses to reaffirming/reconstructing a world of meaning from significant loss alongside other ageing-related challenges (Neimeyer & Holland, 2015). These findings helped us further acknowledge the role of ageing in shaping older people’s experiences of bereavement.

In addition to what has been found in previous research, our study extended the picture of bereavement in later life by paying close attention to ageing and its impacts on older people’s defence from loss. By capturing the older people’s increasing risks of losing capabilities and meaning, our data suggested that ageing could gradually undermine individuals’ defence to draw on varied resources to deal with bereavement (Bolmsjö et al., 2019; Ettema et al., 2010). As a result of losing others and the relational meaning that may be a significant social source for protection, conversely, bereavement could also compound the decline of resilience and the motivation of older people as they age. This fragility of defence was even more pronounced among the older bereaved people with a ‘stiff upper lip’ who have limited abilities to articulate suffering and thus seek support. Whilst these emotional barriers may be rooted in the older people’s earlier life histories, these challenges also reflected how cultural, generational and gender circumstances may smother older people’s defence from bereavement in later life.

While highlighting the impact of ageing, our study also broadened the conceptual scope of bereavement in old age by suggesting such experiences may involve not only relational loss but also the more existential loss of the self (Bolmsjö et al., 2019). Indeed, bereavement did not necessarily give rise to the older people’s existential struggles with loss as clearly evidenced in our data and elsewhere (Holm et al., 2019; Richardson, 2014). However, facing the loss of others whilst experiencing declined personal defence could leave them at a growing risk of losing meaningful resources to affirm their being, as well as confronting the painful reminder of their own mortality. As such, the accumulation of these deeply painful struggles could perpetuate the fundamental fear that older peoples’ own lives, memories, narratives and identity are to be increasingly forgotten, unvalued and irretrievably lost (Sjöberg et al., 2018).

Despite the increased risk of suffering from the existential pain of loss alongside ageing, our chosen context of retirement communities provided a unique setting to examine how the older people sought to retain their defence from a deeper sense of loss and what may contribute to building a buffer. Although not all of our participants moved to retirement-living in direct response to bereavement, many found such community-based environments could provide social resources helping them mitigate the deeper impacts of their loss. Whilst acknowledging the social benefits of retirement-living which may provide a buffer from bereavement, we also noted that a bereavement-supportive culture was still yet to be developed both within the communities and wider society. This poses a challenge to facilitate an ongoing structure to better understand and support older people’s multifaceted loss and bereavement.

Beyond the specific context of retirement-living, the older people in our study also sought varied strategies to retain their resilience and further to ascribe meaning to their loss and bereavement at a more existential level. For some, the best way of dealing with the deeper pain of losing loved ones and the wholeness of the self was to pass on their memories and lives to the younger generation. For others, developing a renewed approach to seeking deeper meanings for the loss of both others and themselves could be particularly meaningful and may even lead to existential growth (Sjöberg et al., 2018). Whilst some old-older people, such as Adam, showed stronger resilience, we found that such protections were not simply a factor of their age but were deeply embedded in their social connections and lived experiences. As such, fundamental to these older bereaved people’s resilience is their access to adequate social channels and their own inner strength. These can help address their existential needs when facing bereavement and its compounded impacts on their ageing being (Ettema et al., 2010). There is clear evidence that experiences of bereavement in later life should be understood and thus adequately supported in the deeper context of ageing that emphasises the loss as both relational (*others*) and existential (the *self*).

## Implications

By illustrating the deeper meanings of bereavement in later life, our study prompted a further question of how to better support older people to retain their defence and seek meaning (particularly existential meaning) to deal with loss. Given the multifaceted and pervasive disruptions of bereavement, a holistic and ongoing support structure is required to empower older people to negotiate for meaning both for their relational and existential loss in the context of ageing. Pivitol to this structure is a ‘grief literacy’ in communities and wider society to enable both older people and others (e.g. family and care professionals) to be more knowledgable and proactively responsive to the unique challenges presented by bereavement in old age (Breen et al., 2020). This grief literacy should also highlight older people’s existential needs, calling for better understandings and support for deeper pains of alienation and meaninglessness when facing the accumulative challenges of bereavement and ageing.

To facilitate such a grief literate culture, local communities and/or retirement villages should play an essential role by facilitating emotional language and compassionate interactions between older (bereaved and non-bereaved) people, families and care professionals. Policy emphasis on education for all community members also lies at the heart of this cultural development process, to proliferate ongoing knowledge and structures to better recognise the deeper needs of those bereaved in later life. This community-based approach could allow for a more sympathetic and inclusive atmosphere, enfranchising older people’s experiences and addressing their concerns about multifaceted loss and access to support moving forward. Given the highly personal and often complex feelings of loss in old age, community-based self-help groups may be a good starting point for propagating grief literacy. This model could provide a non-clinical platform for older people with similar experiences to develop mutual understanding and reciprocity in a more spontaneous and continuous manner. Life/story sharing with trusted others and inter-generational activities may be other avenues to explore, enabling older people to have their meaning-making and life experiences understood and remembered. In addition, it is equally important to ensure professional care (e.g. psychotherapy) is available for those with further needs and difficulties.

## Limitations

The main limitation of this study lies in the lack of ethnic diversity of the interview sample as only three non-white participants were recruited. This racially homogeneous sample may have also prevented us from identifying any significant cultural differences between the participants in the UK and Australia. This limitation resonates with an issue more prevalent in current bereavement research; that is, a lack of accurate samples that reflect the socio-demographical makeup of the broader population. Therefore, future investigations should endeavour to explore older people’s bereavement experiences with a more ethnically diverse sample and/or between more socio-culturally distinctive countries.

Another avenue for future investigations is to research bereavement beyond the current retirement-living context. Although we do not find that this unique context played a decisive role in shaping our findings, the environment in which the residents were situated and their resultant frequent confrontations with loss and/or bereavement may have shaped their naratives. Therefore, it is important to explore and possibly compare our findings to experiences of home-dwelling older peoples (e.g. those living alone or with family). It is also worth examining the bereavement experiences of older people residing in care homes where deaths and deterioration are more prominent and unavoidable.

Finally, given older people are a vastly diverse group of people in different and even competing demographical and physical conditions, further research should explore if and how gender, health, finance and family structures can shape older people’s defence from bereavement and subsequently their deeper pain of loss.
